# Exploring the dynamics of adaptability in the translation of mental health education material: the perspectives from Chinese tertiary students studying in Australia

**DOI:** 10.3389/fpubh.2026.1961587

**Published:** 2026-09-30

**Authors:** Jingyi Yang

**Affiliations:** School of Languages and Cultures, The University of Sydney, Darlington, NSW, Australia

**Keywords:** adaptation theory, Chinese international students (CISs), English-Chinese translation, dynamics of adaptability, mental health translation

## Abstract

**Introduction:**

With technological advances, health communication studies have increasingly focused on multimodality. In contrast, as adaptation has been merged with cross-cultural translation, the dynamics of adaptability have been discussed primarily in theoretical research and other areas of translation, and only a few medical and health translation studies have emphasized this point.

**Methods:**

Using the dynamic dimension in Verschueren’s Adaptation Theory, the current study used a questionnaire survey and semi-structured interviews to explore the dynamics of adaptability in the Chinese translation of mental health education materials for Chinese international students (CISs) studying at Australian universities. Two hundred CISs were invited to take part in this study.

**Results:**

CISs believed they could finish reading the translation within 10 min, but they still preferred more concise translations. Translation length influences the reading strategies CISs select. The participants preferred two forms of translation: (1) the combination of pictures and text, and (2) videos. To improve the linearity of the translation, the former part could be more attractive and motivating. The former and the latter parts of the translation should be integrated to promote dynamic meaning generation. It was also suggested that the links at the end of the translation be incorporated directly into the translation and be clear and up to date.

**Discussion:**

This study applied the dynamics of adaptability to investigate CIS’s view of the Chinese translation of mental health education material and appropriate improvements. It will establish a reader-oriented manifestation of the dynamic dimension of adaptability for relevant research.

## 1 Introduction

Health communication studies have developed in this digital age (1–3), and research on translating mental health education materials requires similar advances. As a communicative action and a transcultural activity, translation can also be studied under the pragmatic framework to facilitate communication among language users (4). Adaptation Theory provided a multi-angled, multi-layered view of translation, which is a special and complex process (5). This theory broadened the research horizon of translation studies and provided it with a coherent, unified framework (6, 7). However, current translation studies in this area seldom emphasize the dynamic dimension. In addition, as an increasing number of Chinese international students (CISs) pursue higher education in Australia (8), their mental health while studying abroad has become a more pressing concern. This study aims to identify the dynamics of adaptability in translating mental health education materials for CISs studying in Australia. Accordingly, we proposed two research questions to fill the gap:

*RQ1*: According to CISs studying in Australia, what should be improved regarding the dynamics of adaptability in the Chinese translation of the mental health education material?

*RQ2*: What strategies could be adopted to solve these matters?

The literature review below will introduce the previous relevant discussion, the research gap, and the theoretical framework of the current research. The research field and the translation material employed will be introduced in the next section. The methodology part contains methods for data collection, processing, and analysis. After quantitative and qualitative analysis, a detailed discussion will be given to provide an in-depth excavation of the results.

## 2 Literature review

Mental health communication towards Chinese migrants and international students has long been discussed in mental health studies. As early as 2008, Blignault et al. (9) conducted in-depth interviews among Chinese migrants living in Australia, and the results identified factors of mental health service access of the group, including mental health literacy, communication difficulties, stigma, confidentiality concerns, service constraints, and discrimination. See et al. (10) sought to explore the factors influencing Chinese migrants in Australia who seek help from general practitioners (GPs). Cultural stigma, practical barriers such as costs, and self-motivation and support from family mattered. GPs required more current cultural competency training. Research on mental health communication among Chinese migrants in other countries has yielded similar findings. In a study among Chinese in the Netherlands, Liu et al. (11) found that communication problems and a lack of knowledge of the health system led to the underutilization of mental health services. For Chinese migrants in the United States, the two most important factors are social stigma and normative beliefs (12).

Studies on mental health communication with Asian young adults are mainly on international students in universities. Jin and Acharya (13) proposed seven themes for developing tailored messages to improve the mental health of Asian international students, such as increasing mental health awareness and reducing stigma, and ensuring resources are available and accessible. Cogan et al. (14) also studied how to enhance mental health communication with Asian international students in the UK, and identified three major themes: negative beliefs, stigma and fear of judgement; adaptation and acculturation difficulties; and communication barriers. Xiong et al. (15) argued that the most significant challenge faced by CISs in Australia was language barriers and communication difficulties. Targeted resources, supports, and interventions are beneficial. These studies have pointed out the factors and improvements that should be made in mental health communication towards Chinese migrants and international students, but they only pointed out the possible factors, such as cultural stigma, and the general actions to take. They did not analyze the detailed changes that should be made in the communication or the education materials.

As the limitations of the fidelity-centric approach have been recognized, the connecting grounds of translation and adaptation have attracted attention in translation studies (16). With several scholars reinforcing the link between these two practices in the 2000s (17–20), theoretical studies have also demonstrated the dynamics of adaptability in translation. Reaching dynamics can help fulfil the purpose of communication (21, 22). Wang (23) noted that translation is both a process and a result of the dynamics of adaptability. Literal translation, free translation, domestication, and foreignization are all translation methods. The dynamic strategy could be followed when selecting the translation methods. Yang (24) reproved Nida’s Dynamic Equivalence by pointing out that Nida’s theory and Verschueren shared similar points. Several studies explored the dynamics of contextual adaptability. Translators could undertake context adaptation in their work; however, they could also recognize that the core of adaptation lies in dynamics. Contextual adaptation in translation is a dynamic process (25–29). There are not many recent theoretical studies that integrate the dynamics of adaptability with translation studies; a potential reason is that previous studies have already discussed existing views, and a new direction is urgently needed.

Numerous researchers have applied dynamic adaptation across various translation research domains, such as literary, publicity, and advertising translation (30–33). However, only a few studies have employed it for medical and health translation studies. Liu (34) applied this theory in medical translation research; Li and Xia (35) and Liang (36) used it in their discussions of traditional Chinese medicine translation. Nevertheless, they stressed only the dynamics of the translation process and failed to apply the detailed dimensions of these dynamics proposed by Verschueren: temporal, contextual, and structural. The research methods in these studies still rely on text analysis, focusing on the source text (ST) and the target text (TT). Considering the gap left by earlier studies on cross-cultural mental health communication and health translation, it is essential to apply all three dimensions to medical and health translation research.

## 3 Theoretical framework: dynamics of adaptability in adaptation theory

Proposed by Verschueren in 1978 and further refined in *Understanding Pragmatics* (2000), the Adaptation Theory is a pragmatic framework for language adaptation across contexts. Verschueren defined adaptability as “the property of language which enables human beings to make negotiable linguistic choices from a variable range of possibilities in such a way as to approach points of satisfaction for communicative needs.” [Verschueren (37), p. 61] Alongside contextual correlations and structural elements of adaptability, Verschueren (37) illustrated the dynamics of communicative adaptation. Firstly, he located three key aspects of dynamics in communication: (1) the temporal dimension, (2) dynamics and context, and (3) dynamics and structure. These aspects aim to demonstrate the relationships among dynamics and time, context, and structure. Meanwhile, Verschueren introduced the strategies of meaning generation and the dynamics of interactive meaning generation. Together, these aspects shape the dynamics of adaptability.

Only a few medical translation studies are conducted from an adaptation-theory perspective (38–41). He (39) performed a translation analysis examining the contextual correlates and structural elements of adaptation. The translation report of Yi (41) is more extensive than He′s, as it incorporated all four dimensions of the theory. Adaptation theory has been applied in medical and health translation; yet, study findings are limited and inconsistent. The theory is only partially implemented in certain investigations, and only a few studies examine the dynamic dimension. To investigate the dynamics of adaptability, additional applied research on medical and health translation is required.

## 4 Research field: translation and adaptation of mental health education knowledge

The Transcultural Mental Health Centre (TMHC) is a statewide service established in 1993. It collaborates with health professionals and communities throughout New South Wales to promote positive mental health among individuals from culturally and linguistically diverse communities (42). Among the multilingual general mental well-being resources provided by TMHC, we chose *Building Resilience* and its Chinese translation as the research material for this study. Resilience refers to “the ability to bounce back from stressful or challenging experiences” (43). This topic aligns with the current situation of CISs, who are facing increasing mental health problems when studying abroad. The Chinese translation is 4 pages long, allowing participants to read it in about 10 min. Meanwhile, this old material is still in use more than 10 years after its publication. We also adopted it to test what could be improved in the outdated mental health education information and its translation in other parts of the project.

## 5 Research design

This study aims to get the following results to help translators make their choice: (1) the general situation of dynamics in CIS’s reading, (2) the differences among the participants and their suggestions on improving the translation in terms of the dynamics of adaptability. Thus, we employed a mixed-methods design, and the research consists of two stages. In the first stage, a 5-point Likert-scale questionnaire with 5 questions on the dynamics of adaptability was developed to collect quantitative data describing the dynamics during CIS’s reading. In the second stage, semi-structured interviews with three groups of questions, developed based on the results of the quantitative analysis, were conducted, yielding detailed and diverse suggestions for follow-up. While the quantitative questionnaire surveys were conducted before the qualitative interviews, the latter carry more weight than the former. Qualitative analysis is given greater weight than quantitative analysis.

Three questions were proposed on “locating” the dynamics of communication. Questions 1, 2, and 3 stress the three aspects Verschueren (37) mentions: (1) the temporal dimension, (2) dynamics and context, and (3) dynamics and structure. The dynamics of the communication are closely linked to time. Time, or the temporal dimension, provides the raw material for communicative dynamics. The stages of language development, memory, and time planning all belong to the temporal dimension. “It is difficult to read this translation in 10 minutes” is designed to assess whether participants can finish reading it within a time limit. Dynamic context is the second aspect of the dynamics in communication. Social relationships are dynamic, personal states of mind are dynamic, and different forms and linguistic channels lead to different dynamics. Question 2, “I prefer another form of translation to a written form” investigates whether Chinese students nowadays still prefer the traditional written form of knowledge, as different forms provide different kinds of dynamic context. A third aspect of the dynamics of communication is the dynamic structure, and its basic property is linearity. The linearity of language holds across all levels of the text, including word order, sentence order, and information structure. The former part of the translation affects the reading experience of the following parts. The linearity is analyzed from Question 3: “The information in the previous text did not make me want to follow the instructions in the following text”.

Regarding the strategies involved in dynamic meaning generation, Verschueren (37) mentions the interplay between explicitness and implicitness. Question 4, “The knowledge in the translation was not subtle enough and caused discomfort”, was added to the questionnaire. The implicitness of the translation can ensure that the students, especially those students with mental pressure and mental disorders, do not have more bad feelings or frustration during the reading. In terms of the dynamics of adaptability, Verschueren (37) lastly analyzes the dynamics in the interactive meaning generation. The target readers’ reactions also influence the generation of meaning for the following information. Although the participants did not speak with the writer or the translator, they did respond to the knowledge provided invisibly, which may have influenced comprehension of the latter part. “The latter part of the translation contains unnecessary information that has been explained before” was designed to find out whether the arrangement in the translation suits the participants’ comprehension process (see Table 1).

**Table 1:** Questionnaire.

Dynamics of adaptability	Questionnaire
“Locating” the dynamics of communication	Q1. It is difficult to read this translation in 10 min. Q2. I prefer another form of translation to a written form. (e.g., oral, video) Q3. The information in the previous text did not make me want to follow the instructions in the following text.
Strategies of meaning generation	Q4. The knowledge in the translation was not subtle enough and caused discomfort.
The dynamics of interactive meaning generation	Q5. The latter part of the translation contains unnecessary information that has been explained before.

The questions for the semi-structured interview were developed based on the results of the quantitative analysis. “Can you finish reading this translation within 10 minutes?” and “This translation spans four pages. How long do you think is appropriate?” were developed from the first item in the questionnaire. The second item in the questionnaire, which investigated the most suitable form of mental health translation, was revised to: “When you are in a hospital or clinic, or reading similar mental health knowledge on your mobile phone, which do you prefer, text, picture, or video?” The third question in the interview, “After you finish reading this translation, will you visit those websites and read the books and materials listed at the end of it?”, corresponds to both Questions 3 and 5 in the questionnaire. This question seeks to investigate the relationship between the former and the latter parts, that is, what influence the former has on the latter. This can be either motivation or unnecessary content that has already been explained. According to the quantitative results, most participants reported that the translation was subtle and did not cause any distress, while only a few disagreed, and they were not willing to take part in the interview. Further research could be conducted with these special participants to understand why they felt unwell while reading the translation and to identify strategies to better meet the needs of special readers (see Table 2).

**Table 2:** Interview questions.

Dynamics of adaptability	Interview
The Temporal Dimension	Q1. Can you finish reading this translation within 10 min? This translation spans four pages. How long do you think is appropriate?
Dynamics and Context	Q2. When you are in a hospital or clinic, or reading similar mental health knowledge on your mobile phone, which do you prefer, text, picture or video?
Dynamics and Structure The Dynamics of Interactive Meaning Generation	Q3. After you finish reading this translation, will you visit those websites and read the books and materials listed at the end of it?

### 5.1 Data collection and processing

Before data collection, a pilot study was conducted in a Chinese studies class. We provided paper questionnaires to the students in the class. There were 30 students in the class, and 20 students finished and submitted the questionnaire to the researcher. The problem revealed in the class was that the initial questionnaire did not make it clear what the numbers on the Likert scale referred to. “Agree” and “Disagree” should be added above the Likert numbers. Expert review was conducted by two experts: one in translation studies and the other in second-language learning. After discussing with them, I kept the original items but revised the Likert numbers.

We invited 200 CISs studying in Australia via convenience sampling through two channels: classes taught by Chinese studies teachers and the university library. The Chinese studies classes primarily consist of Chinese undergraduates seeking to advance their education in diverse fields, such as Chinese literature. Other participants who were invited in the university library encompassed a wide range of students, including undergraduates, postgraduates, and PhD students. Some of them were psychology students who contributed substantial professional knowledge in their responses. By recruiting a diverse array of participants, the two pathways enabled us to obtain comprehensive research data. Of the 200 invited participants, 182 students answered all questions, and questionnaires with missing data were deleted. The demographic information of these participants is in Table 3. Of the participants, 47.87% agreed that they need the Chinese translation of mental health knowledge, with 18.7% strongly agreeing and 29.1% agreeing. While 30.2% of the students held a neutral view, only 22% expressed they did not need the Chinese version. Although participants have a certain level of English proficiency, the translation of mental health materials is still required.

**Table 3:** Demographic information of participants.

Variable	Group	Frequency	Percent
Gender	Female	125	68.7
	Male	57	31.3
	Total	182	100.0
Age	20 and below	55	30.2
	21 to 30	125	68.7
	Over 30	2	1.1
	Total	182	100.0
Time studied in Australia	1 year or less	66	36.3
	1 to 2 years	65	35.7
	2 to 5 years	38	20.9
	More than 5 years	13	7.1
	Total	182	100.0
Education level	Bachelor	117	64.3
	Master	63	34.6
	PhD	2	1.1
	Total	182	100.0
Language level	IELTs	113	62.1
	None	39	21.4
	TOFEL	11	6.0
	PTE	10	5.5
	Others	9	4.9
	Total	182	100.0
Needs for translation of mental health knowledge	Strongly agree	34	18.7
	Agree	53	29.1
	Neutral	44	30.2
	Disagree	39	16.5
	Strongly disagree	10	5.5
	Total	182	100.0

In the first stage, data were collected using both offline paper questionnaires and online questionnaires via Qualtrics. Written consent was collected before data collection. Chinese version of the items was used to make the questionnaire more accessible. The online and paper versions of the questionnaire are identical, and the participants can choose the form they like. The paper questionnaires were provided because the PDF translation from TMHC is hard to read on phones due to its dense text. 162 participants completed the hard-copy version, while 20 chose to answer online, indicating that the paper questionnaire was preferred by the CISs.

In the second stage, we invited participants from both participants invited in classes and those who completed the survey in the library. This can ensure that the interview participants can provide diverse data. 20 participants expressed interest at the end of the questionnaire and finally took part in the interview. Both face-to-face and Zoom interviews were offered to participants, and all 20 CISs chose Zoom. The interviews lasted about 20–30 min and aimed to gather their views on the translated passage concerning the dynamics of adaptability. To avoid the language barrier, the interviews were conducted in Chinese. If the participants provided brief answers, we asked for potential reasons to obtain more detailed data. Audio recordings were collected during the interviews and were transcribed for qualitative analysis.

### 5.2 Data analysis

We first transcribed the data from both paper and online Qualtrics questionnaires into a Microsoft Excel form and conducted a second check before using it for analysis. The quantitative data were then imported into SPSS 29.0. Descriptive statistical analysis, including mean scores, standard deviation, frequencies, percentages, medians, and interquartile ranges, was conducted to examine the five items. The results provided a general reflection on the participants and served as the foundation for developing the interview questions.

The interview responses were transcribed in Microsoft Word for thematic analysis. There were some errors in the transcript due to vagueness, hesitation, and repetition, so we corrected them to ensure data quality. Google Translate was used to translate the interview responses from Chinese to English to improve efficiency, and we conducted post-editing of the machine translations to ensure that culturally and pragmatically significant expressions were verified. The English version of the interview responses was allocated to the interview questions and then entered into NVivo 15.1, because we had to ensure that the translation of the codes fit the original context. Reflexive thematic analysis was adopted because a single researcher completed the inductive coding process. After coding the transcripts of each question, I summarized subthemes and themes (see Appendix I) for each interview question and then reviewed them to ensure the quality of the analysis. Representative divergent cases were added as a theme, while the less crucial ones were deleted.

## 6 Findings

### 6.1 Findings from quantitative analysis

#### Descriptive statistics

Descriptive statistics were analyzed prior to developing the interview questions for qualitative thematic analysis. To demonstrate the general situation, distribution, and outliers of the items, mean scores, standard deviations, frequencies, percentages, and boxplots were used to provide a comprehensive description.

In Table 4, the mean scores and standard deviations of the five questions are presented. Questions 1 to 3 exhibit a higher standard deviation than the other two questions. Among these three questions, Question 2 has the largest average and standard deviation. The average score for this question is around 3, with a standard deviation of 1.096. This indicates that participants held diverse views on the preferred forms of translation. Most students could complete the reading in 10 min, as indicated by a mean score of 2.23 on Question 1. Only a few of them said they could not. The outcome of Question 3 matches that of Question 1. The participants largely concurred that they would like to adhere to the instructions provided after the translation, with little disparity between them.

**Table 4:** Mean score and standard deviation.

Questions	Mean	Std. deviation	*N*
Q1	2.23	0.985	182
Q2	2.96	1.069	182
Q3	2.65	0.950	182
Q4	2.01	0.824	182
Q5	2.53	0.920	182

Questions 4, “The knowledge in the translation was not subtle enough and caused discomfort”, and Question 5, “The latter part of the translation contains unnecessary information that has been explained before”, concentrate on strategies for meaning generation and the dynamics of interactive meaning generation, respectively. The standard deviations are the lowest across all five questions in this section. On meaning generation, the participants indicated that the translation was subtle and did not provoke any discomfort. The mean score of 2.53 and a standard deviation of 0.92 for Question 5 suggest that Chinese students do not see the instruction portion of the translation as containing excessive knowledge that had already been explained.

Table 5 demonstrates the frequency and percentage for each item in the questionnaire, and the box plot in Figure 1 shows the median, interquartile ranges, and the outliers. Responses to Question 2 were widely distributed, and the median was 3. With 35.7% of participants choosing 1 or 2 and 33.5% choosing 4 or 5, this item shows a strong disagreement among participants. Question 4 received the lowest mean score of 2.01, as analyzed above, with 7 participants (7.8%) selecting 4 and 1 participant selecting 1. The responses concentrated around 1 and 2, with a median of 2. Participant 186, who chose 5 for this item, is also marked as an outlier on two other items. The other three questions, Questions 1, 3, and 5, showed a similar pattern: more than 50% of responses fell between 2 and 3. While the median of Question 3 is 3, the medians of the other two questions are both 2.

**Table 5:** Frequency and percentage.

Questions	Items	1	2	3	4	5
Q1	Frequency	42	82	38	15	5
	Percentage	23.1	45.1	20.9	8.2	2.7
Q2	Frequency	15	50	56	49	12
	Percentage	8.2	27.5	30.8	26.9	6.6
Q3	Frequency	20	59	74	23	6
	Percentage	11.0	32.4	40.7	12.6	3.3
Q4	Frequency	51	88	35	7	1
	Percentage	28.0	48.4	19.2	3.8	0.5
Q5	Frequency	22	70	64	23	3
	Percentage	12.1	38.5	35.2	12.6	1.6

**Figure 1:**
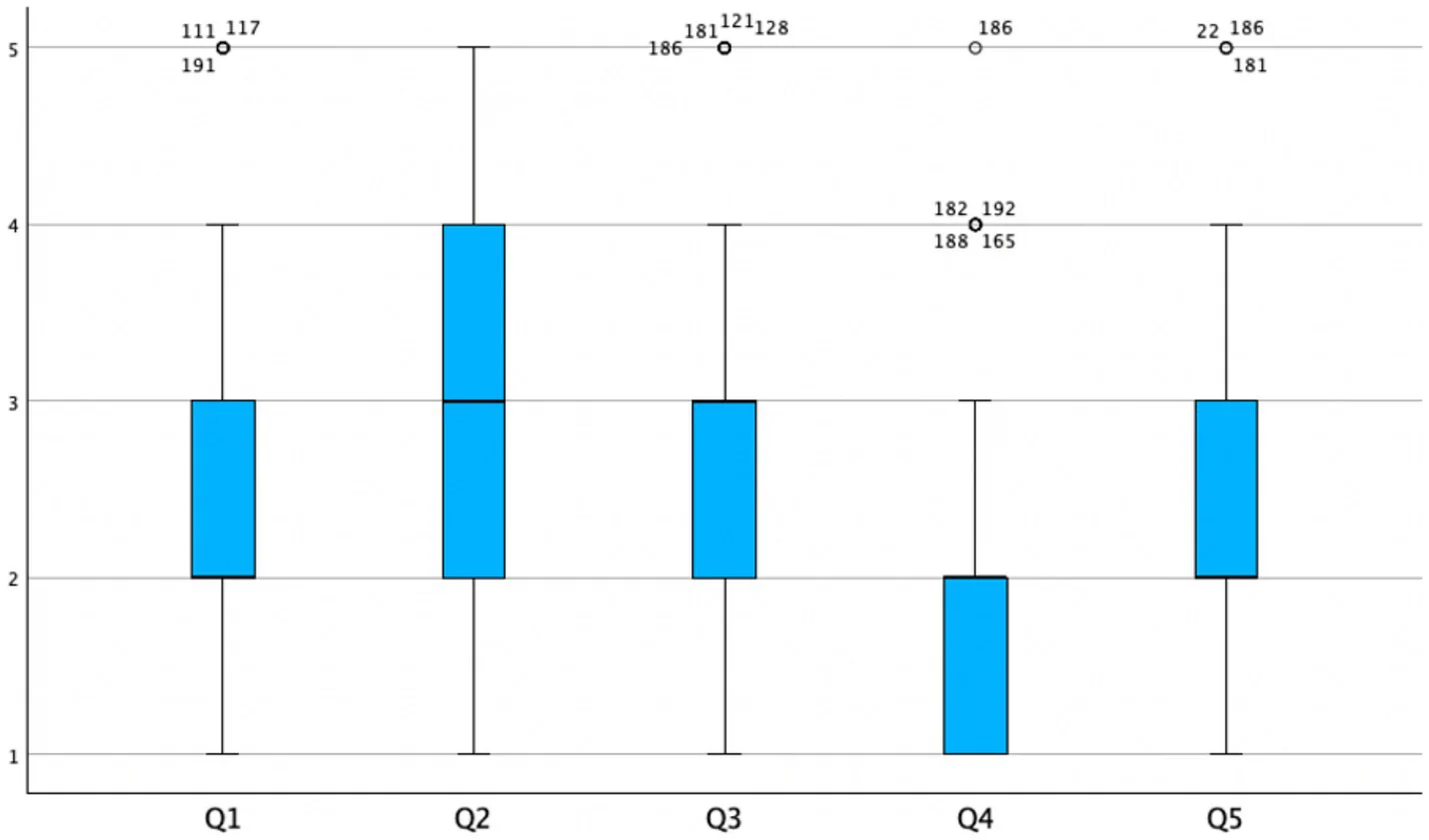
Box plot.

### 6.2 Findings from qualitative analysis

#### 6.2.1 The temporal dimension of dynamics

Question 1, “it is difficult to read this translation within 10 minutes,” was included in the initial set of interview questions. Students were presented with two questions regarding the temporal dimension of dynamics: “Can you finish reading this translation within 10 minutes?” and “This translation spans four pages. How long do you think is appropriate?” As far as CISs are concerned, the time required to read the translation depends on their reading ability and strategies. A long translation made participants less patient, and a concise version is more suitable for digital reading. The length of the translation also influenced their choice of reading strategy and information processing.

##### 6.2.1.1 Reading time

###### 6.2.1.1.1 The Reading time of translation depends on target readers’ Reading ability

Some participants held that reading proficiency can affect their reading duration, as Participants 6, 7, 16 and 4 described,

*Well, the length is suitable in Chinese, but I might not finish the English reading. (Participant 6).*

*My reading speed is actually rather sluggish, so 10 min is ideal for me. (Participant 7).*

*Yes, I have read it, and it took me about 7 min to finish. (Participant 16).*

*I think it’s okay because I’ve been reading a lot of literature lately. I think it’s fine; four pages is not excessive. (Participant 4).*

Participants’ reading abilities have varying effects on their reading. English ability restricted Participant 6 from reading the English version of such content. Participant 16 can complete the reading in 7 min; however, Participant 7 required 10 min due to a slower reading pace. Reading training also enhances one’s reading proficiency. Having recently studied extensive literature, Participant 4 did not consider the translation excessively long, as he was accustomed to reading substantial texts. Language ability, reading speed, and reading training all influence participants’ reading time.

###### 6.2.1.1.2 The target readers’ reading strategies influence their reading time

The reading strategy students select also influences how much time they spend reading. Participants 5, 3 and 12 mentioned this point in their responses:

*Yes. I believe I should be able to do it. I’ll have an overall impression after reading it. (Participant 5).*

*Well. This length. I believe it will take me around 8 to 9 min to read this. Because there are several big sentences, I may read it slowly to avoid errors. (Participant 3).*

*Yes. It depends on how carefully you read it. If it’s like a flyer and you do not read it very carefully, it should take only about 5 min. However, if you read it thoroughly and want to absorb the knowledge, it may take longer. (Participant 12).*

After browsing the translation for such a brief period, Participant 5 could form a general impression. Participant 3 recommended a duration of 8 to 9 min, as he desired to allocate additional time to the lengthy sentences in the material to prevent errors. Participant 12 employed the opposite approach, focusing only on the primary information. If one does not read attentively, they will spend approximately 5 min on the translation, as certain components, such as the references, may be disregarded. Therefore, the reading strategies of target readers also influence their reading time; the translator could investigate these strategies and then tailor the translation accordingly.

##### 6.2.1.2 Appropriate length

###### 6.2.1.2.1 Long translation of mental health education knowledge makes CISs less patient

The initial reason is that the translation is so lengthy that it causes CISs to feel less patient. Participants would lose patience if the material exceeded two pages:

*Two or three pages. Two to three pages is not excessively long. Yes, if the content is too long, I will lose patience and stop reading. (Participant 15).*

*I feel that it is too long and should be reduced. I think it should be reduced to half. It is more reasonable to reduce it to half. In general, I believe two pages of a flyer, front and back, are sufficient. If I must read another piece of paper, I will be less patient when reading it. (Participant 1).*

The optimal length of the material, as suggested by participants, is two pages, with one or two pages recommended by Participants 15 and 1. “Two pages on one page of fly, front and back” will not cause readers to lose their patience.

###### 6.2.1.2.2 The participants would not like to read long translations they are not interested in

An additional reason why students desired shortened reading materials is that they did not find the content of the translation to be appealing, as Participants 14 and 9 mentioned:

*[…] If it is too long… How should I put it? Unless I am very or highly interested in it. I will find it boring to read and will stop. (Participant 14).*

*It is indeed not attractive enough to me. It is not attractive enough. I think two pages may be the upper limit for content that I am not very interested in. I am not willing to spend too much time on content I am not very interested in, and that does not benefit me much. (Participant 9).*

Students found mental health knowledge tedious. The content of the translation was not appealing to Participants 14 and 9, and they believed that it should be limited to one to two pages. They would continue reading lengthy materials only if they found the content very attractive. Less complex material is visually appealing and could captivate the readers’ attention. Detailed and professional knowledge is not necessary for this type of material.

###### 6.2.1.2.3 Concise translation is preferred for digital reading

With the advancements in technology and media, digital reading has become increasingly prevalent, particularly among young people such as university students. While mobile phones facilitate digital reading, individuals are reluctant to dedicate excessive time to reading in such a fast-paced world. The translation must conform to contemporary reading preferences. As Participants 5 and 13 regarded,

*Ah, if you read it on your phone, I feel that about 4 pages on your phone is suitable, but the content should be more concise. Or about two pages on the paper. Um. When I read it on my phone after opening it, and saw a lot of small words, it made me a little disappointed. I felt that it was troublesome, right? Um. It is not that it is useless. Simply glancing at a lengthy string of words made me feel that I did not want to read it. (Participant 5).*

*About three pages, or WeChat can load all at once. Because, in my opinion, being short and concise is preferable. Oh, I might really enjoy it if the content is better. (Participant 13).*

The arrangement of information on the phone significantly differs from that on the paper fact sheet. The compact, dense format on mobile devices may discourage readers from accessing the content, as noted by Participant 5 in his response. The text on the phone could be concise, using larger and less condensed words. Participant 13 recommended that the translation length allow for simultaneous information loading. The prevalent trend of digital reading has led CISs to favor brief and succinct texts, characterized by larger and less condensed words.

###### 6.2.1.2.4 The length of translation influences the Reading strategy and information processing

As the length of the content affects the reader’s comprehension, readers will also adapt their reading strategies according to the material they are going to read. Additionally, these have a substantial effect on information processing and comprehension. Readers will opt to read the content they need rather than the entire text when presented with a lengthy text. As presented by Participant 9,

*When reading this four-page essay, the reader may prioritize the subject that interests him. For example, if I am aware that I have a problem with resilience, when I read this essay, I might neglect the introduction on resilience. How is resilience defined in such an academic setting or the field of mental health? What environments will it form in? Which environments will it be most effective in? I may not read these. I’ll only look at the final step. I’d like to find a way to contact you, such as a phone number or an email address. (Participant 9).*

Readers will automatically select the content that is most helpful for them due to the limited time available. Participant 9 suggested that individuals with psychological issues should review the methods in the translation, as well as the contact phone number and email address provided. The definition and the reason may be disregarded as they are less practical. Apart from reading selection, how readers comprehend the content within a limited time depends on their reading training.

*Yes. I think it is fine to have four pages. The key is to control the reading time of each section. I think one section should be about 1 to 2 min long. This will motivate me to keep reading and finish it. If it is about 10 min long and each section is relatively short, then after I finish reading this section, I will move on to the next. In this way, I will feel that it is not that long. Then the next section is new. I can try harder and encourage myself. (Participant 7).*

Participant 7’s reading method involves dividing the text into multiple parts and allocating time to each section. Dividing the reading into multiple assignments can motivate him to persist and make the reading feel shorter. In the Chinese education system, students are instructed to read both Chinese and English texts with various approaches. The time constraint compelled them to employ the strategies they had learned in class.

#### 6.2.2 Dynamics and context

Question 2 in the questionnaire, “I prefer another form of translation to a written form,” received the highest mean score of 2.96 and a standard deviation of 1.069 among the five items. The findings indicated that a portion of the students were dissatisfied with the current written form of mental health reading material. The second question of the interview aimed at assessing the impact of the translation’s form on the reader’s comprehension: “When you are in a hospital or clinic, or reading similar mental health knowledge on your mobile phone, which do you prefer, text, picture or video?” As far as the responses mentioned, students taking part in the interview preferred (1) the combination of word and picture, as well as (2) the video.

##### 6.2.2.1 Combination of pictures and text

###### 6.2.2.1.1 The combination of text and picture makes participants’ Reading fast and clear

The second reason for preferring the text-picture combination in Chinese translation is that it facilitates rapid and effective reading of CISs. Videos, on the other hand, will be displayed for a definite duration. As Participant 11 described,

*Combine text with images. Pictures can be more concrete, whereas words can explain things in greater detail. When it comes to videos, it will undoubtedly take considerable time to explain everything. If you want to be concise and clear, I believe you should merge images with words. (Participant 11).*

Images accompanied by words are more concrete and detailed, yet require less time than videos. Participant 3 expressed similar feelings, noting that images accompanied by text required less time and increased his willingness to read the translation, as they helped him grasp the key idea more effectively. He also suggested that for a two-page translation, three or four images were appropriate:

*However, if it includes pictures and text, I can quickly grasp the main points, and I will be more motivated to read it because it takes less time. Well. I believe that three or four images will do for this two-page piece. (Participant 3).*

###### 6.2.2.1.2 Pictures fit the participants’ digital reading habit

A third reason participants favored picture plus text was that this format accommodated the needs in the digital era. Participants 5 and 13 referenced this point in their responses:

*Oh, I prefer text plus pictures. Yes, I think text plus pictures is more suitable for reading in fragmented time. And, yeah, videos cannot be open all the time, so I may choose to ignore them. (Participant 13).*

*If I were in a clinic, I think I would be more inclined to text. Maybe pictures are better on mobile phones because there is limited space. Also, I find it difficult to read if it includes a lot of words. If I were to read it in its entirety, I would feel a bit annoyed halfway through and would not want to read it anymore. (Participant 5).*

As stated in the linguistic channel section, Chinese foreign students get mental health knowledge through online browsing and from their university. It is essential to modify the translation to align with these linguistic channels. Video is not an ideal medium for Participant 13, as it cannot be watched in a short period. However, he can read images in a brief amount of time. Participant 5 is more likely to select images on mobile devices because they are better suited to the limited space of the phone screen. Too many small words on the screen make people annoyed.

##### 6.2.2.2 Video

###### 6.2.2.2.1 The participants felt that videos are more convenient and easier to understand

Some participants favored videos in that they were more convenient and easier to understand. Participant 2 remarked in his response,

*Um, videos. Yes. Because I believe most students, including my friends and international students, prefer to watch videos rather than read. Videos are undoubtedly more convenient because they can be listened to at any time, regardless of what you are doing. (Participant 2).*

Videos delivered greater convenience as they could be watched at any time, regardless of what the audience was doing, as mentioned by Participant 2. Videos integrate written and auditory stimulus, allowing the audience to select their preferred mode of reception. Participant 18 found videos easier to understand,

*Yeah. Videos, I believe, are required, combining actions in a process and showing how to get from A to B. This type of procedure must be seen so that I can remember it more firmly and comprehend it better. (Participant 18).*

Participant 18 explained that videos illustrated the actions in a process, thereby enhancing his memory for the material and facilitating his comprehension. The participants agreed that the auditory and visual knowledge made videos a convenient and straightforward option.

###### 6.2.2.2.2 Videos create stimulus and passive reception is preferred by CISs

Incorporating the Chinese version of mental health education into videos boosts their popularity. Participant 9 voiced this perspective:

*Well, video is the first. Text comes second, followed by pictures. If someone is present in the video, they will speak to you. If no one is there, you listen to the auditory response. Your visual response is a prevalent stimulus. You also get a visual impression from text. However, you are asked to see it, and you do not read it aloud. You cannot hear it with your ears; instead, you just think about it. (Participant 9).*

Participant 9 determined that videos evoke a response in the audience by providing both auditory and visual stimuli. As for the text, readers must read it actively and contemplate it in their minds, without any auditory or visual stimuli. Consequently, these students were more inclined to view videos. Meanwhile, CISs were more inclined to watch videos because they favored passive reception. As Participants 15 and 19 mentioned,

*Well, I prefer videos because videos are the easiest to absorb. You do not have to actively absorb them. I can absorb them passively. (Participant 15).*

*I prefer videos because text is an active reading process, and unless I really want to read, I will not. Pictures are semi-active because I can look at some graphics and quickly understand their meaning. Videos are purely passive reception, and I will follow them all the way. If the video is interesting, I will finish reading it. (Participant 19).*

Both participants used the terms ‘active’ and ‘passive’ to characterize the reception process. Text reading was regarded as an active process, whereas viewing videos is a passive reception process in which viewers can swiftly access and finish the content. Passive reception made the reading less exhortatory, which made the translation more acceptable.

#### 6.2.3 Dynamics and structure and the dynamic of interactive meaning generation

To examine the linearity of the material in the translation, we included the item “The information in the previous text did not make me want to follow the instructions in the following text” in the questionnaire. “The latter part of the translation contains unnecessary information that has been explained before” was used to test the dynamic of interactive meaning generation of the translation. Both questions aim to investigate the influence of the former part of the translation on the latter. Therefore, we converted them into an interview question: “After you finish reading this translation, will you visit those websites and read the books and materials listed at the end of it?” Some CISs would follow the instructions only if the former part were interesting, but some participants did not realize they needed the knowledge in the translation. Meanwhile, the TT readers would like to have clear, direct, and up-to-date instructions at the end to follow.

##### 6.2.3.1 The former part

###### 6.2.3.1.1 The participants would like to follow the instructions if the former part is interesting

Two of the participants expressed that they would like to follow the instructions if they were interested in the content in the former part. As Participants 7 and 13 revealed,

*Yes. This is more of a matter of interest. When I have completed the reading and find it to be quite captivating, or if I am interested in learning more, I will visit the website. More often, I’m motivated by curiosity and want to see what it’s like. For example, if I want to know more about a specific aspect or some interesting titles, I may look it up. (Participant 7).*

*Yes. As I said, I have probably seen it two or three times. I am curious about it. (Participant 13).*

Participant 7 indicated that he would visit those websites if he thought the content was highly interesting. He added, however, that he would be inspired to learn more if the title aroused his interest. Having read the translation two or three times, Participant 13 would also like to follow the instructions because of curiosity. When translating the material into Chinese, the translator could include captivating knowledge to enhance the linearity’s impact on the readers.

###### 6.2.3.1.2 The participants did not realize they needed the knowledge in the translation

Different readers have varying motivations for reading mental health education knowledge. Although the readers of the ST may seek assistance when they encounter mental health issues, the TT readers, CISs, lacked comparable motivation. The content in the former section is less targeted and requires motivational content, as several participants reported they would not adhere to instructions to visit the websites or read the material at this time due to a lack of demand. As Participants 16 and 19 mentioned,

*No, because I was not asked to read this. To put it another way, if I were in an incredibly difficult circumstance right now and saw someone’s advice below, I might read it, but I have not yet, thus I’m unlikely to read it. Um. (Participant 16).*

*To be honest, I do not have any mental problems in this area now, so I will not read them. If I have, it should be very helpful. (Participant 19).*

These two students encountered no difficulties while reading the translation. Nevertheless, they emphasized that they would adhere to the instructions and might find them beneficial if they had such a requirement. These findings indicated that CISs were unaware of the significance of mental health education in their daily lives. They only seek assistance after they have discovered that the mental issues have already existed, and that the translation did not motivate them since most of them did not have mental health problems at the current stage. The translation could emphasize the significance of acquiring relevant knowledge and preventing mental health issues in daily life. It could include more introductions or examples that are close to real life when studying abroad.

##### 6.2.3.2 The latter part

###### 6.2.3.2.1 The participants prefer direct, clear, and new instructions

In addition to the motivation from the previous section, the instructions could be straightforward and clear. Participant 15 explicitly stated that he would not follow the instructions after the translation, as he believed they contained an excessive amount of content and did not consider them to be necessary:

*Uh. Probably not. Because I do not think it is necessary. And there’s simply too much information. I do not want to read it again after finishing it. I do not want to read it anymore. (Participant 15).*

Participants 10 and 12 mentioned their recommendations that the instructions at the end of the translation could be direct and clear:

*Well, probably not. First, if I go for a consultation, I am unlikely to read the piece from beginning to end. Then it’s too hard because it does not provide a direct help method, and I have to search the website again. (Participant 10).*

*Well, I will not. How should I put this? I do not want to spend time visiting these websites. However, if some of them are about organizing a night out with friends and you include a link in the back, such as a link to register for an event, I may click on it. Hmm. For example, the contact information at the bottom of the piece is too traditional and will not entice me to click on it. That’s true, but if there are some interesting snippets in the article and a link at the end, I might click it. (Participant 3).*

*Well, no, I might pick a few of them. For example, if there is a specific website, I might click on it. Well, because it is more convenient for me. (Participant 12).*

Participant 10 favored the “directly help method,” which implied that he did not wish to conduct another website search. He found websites and literature to be exhausting. Participant 12 desired access to specific websites that were more convenient for him. Participant 3 preferred a link to register for an event over websites that provide mental health knowledge. The methods in the translation were more accessible than the knowledge provided in the link. If links were appended after the translation, they could have been practical, preferably as event registration links rather than websites laden with excessive material. The links at the end of the translation could not be overloaded with excessive material. Instead, they ought to endorse the content presented in the preceding section. The information at the end of the material was also criticized by Participant 9 for being outdated:

*Well, I just feel like I will not click because I do not need it and was not interested in it. Another point is that this article was published in 2010, and all its information, uh, is from 10 years ago, and some is from the 1980s, if I remember correctly. So, for me, it’s not very timely. So, maybe I will learn more about some of the latest topics. (Participant 9).*

Participant 9 noted that the translation employed for the present research was published in 2010. Furthermore, several referenced websites and publications were from the 1980s. This material could offer the latest and most relevant content that more effectively meets students’ needs. These three participants proposed that direct, straightforward, and novel instructions could more effectively engage CISs.

## 7 Discussion

Question 1 in the questionnaire and the first question in the interview provide quantitative and qualitative results for the investigation on the temporal dimension of the selected translation. Although most students could complete the reading in 10 min or less according to the quantitative results, the findings in the interviews partly align this point. Most participants could complete the reading in 10 min, and their reading time was influenced by their language proficiency, reading speed, and the reading instruction received and their reading strategy. Even though CISs were eager to apply the reading skills they had developed, they preferred concise and rapid translations for their digital reading. The translator could offer students shorter content and consider their reading skills and methods when translating the content into Chinese.

Regarding dynamics and context, both Question 2 in the questionnaire and the second question in the interview were proposed. The quantitative results revealed that some participants favored alternative translation forms, such as video and pictures, rather than plain text. The thematic analysis further revealed the forms CISs prefer and the reasons for the preferences. Some participants reported a preference for video or a combination of text and images. They thought pictures allow for faster, clearer reading and are better suited for screen reading. Others proposed that videos were easier to grasp., more convenient, and more appealing to audiences because they generate auditory and visual stimuli. There is a pressing need for new forms of translating mental health education knowledge. Pictures and videos could be used to satisfy various needs. Multimodality could be considered and integrated into the translation of mental health knowledge, and intersemiotic translation could be a new research focus in this translation area. The framework of selection, labelling, and reception (44) could be applied when translating mental health knowledge into other forms.

Results of Question 3 in the questionnaire indicated that most students would like to adhere to the instructions provided in the latter part of the translation. According to the interview responses, however, students said they would like to follow the suggestions if the former part is interesting and arouses their curiosity. Others expressed that they were not motivated to continue exploring the links by reading the former section since they did not have mental health problems. To improve the linearity of the translation, the translators must enhance readers’ interest and convince them of the importance of reading mental health knowledge in the former part.

According to the quantitative results of Question 4, most participants reported that the translation was subtle and did not cause any distress, while only a few disagreed, and these responses are marked as outliers in the box plot. We reviewed the questionnaires from these participants and found that none expressed interest in participating in the interview. The only participant who chose 5 is Participant 186, who was also marked as an outlier on some other items. One possible reason is that this participant did not answer the questionnaire carefully. The reasons for other special readers who were not satisfied with the translation’s implicitness warrant further research.

Question 5 in the questionnaire was designed to investigate interactive meaning generation in translation, and the answers indicated that the participants did not believe the latter section contained many extraneous details that were already covered. In contrast, the qualitative findings in the third group of questions in the interview indicated that the participants did not think they needed the knowledge in the translation since they did not have any mental health problems. The students also recommend that the links in the later section be more direct, clearer, and up to date. It can be concluded that the link between the former and latter part is rather weak, so that the dynamics of interactive meaning generation was not met in the translation. This drawback should be addressed in future translations.

The dynamics of information reception in translation reading analyzed in this study enhanced the previous research by extending the application of the dynamic dimension of adaptation theory to mental-health translation by incorporating target-reader perceptions and reported reading design. Meanwhile, by employing Verschueren’s framework, the current study discussed several aspects of the dynamics of adaptability in detail rather than addressing them in general or merely mentioning them. Last but not least, this study further integrated adaptation with health translation studies and provided a potential direction for multimodality in this area. We are accustomed to digital reading in this digital age. The reader’s cognitive status is influenced by the content, length, difficulty, and form of the translation, as well as their reading strategies. The findings of the current study will improve the translation of mental-health knowledge for CISs.

## 8 Conclusion

This study explored the dynamics of adaptability in translating mental health education materials for CISs. Both quantitative and qualitative analyses have been conducted, during which crucial factors for improving translation, such as length, form, and meaning generation, have been identified, and strategies for improving the translation have been given. The limitations of the study are the use of a single material due to time constraints in the questionnaire survey and interviews, conducting convenience sampling within the university due to limited research support, and the researcher’s interpretation of qualitative data. The implicitness of mental health knowledge translation for special readers warrants further investigation. More studies could be conducted by using health materials in other areas and among other culturally and linguistically diverse groups in the future.

## Data Availability

The original contributions presented in the study are included in the article/Supplementary material, further inquiries can be directed to the corresponding author.
