# Supplementary material for: Exploring the dynamics of adaptability in the translation of mental health education material: the perspectives from Chinese tertiary students studying in Australia
Source: Front Public Health. 2026 Sep 30;14:1961587. doi: 10.3389/fpubh.2026.1961587 (PMC13661939; doi:10.3389/fpubh.2026.1961587)
Supplement: Supplementary file 1 [file Table_1.DOCX]

*Appendix I: Thematic Content Analysis*

| Theme | Sub-Theme | Code | N | Supporting Quotations |
| --- | --- | --- | --- | --- |
| Reading Time | The reading time of translation depends on target readers’ reading ability. | Sure, definitely, ideal, yes | 19 | 10 minutes is ideal for me. (Participant 7) |
|  |  | OK, fine, suitable, | 6 | I think it's okay because I've been reading a lot of literature lately. I think it's fine; four pages isn't excessive. (Participant 4) |
|  | The target readers’ reading strategies influences their reading time. | Carefully, thoroughly, slowly  Avoid error, absorb the information | 6 | Because there are several big sentences, I may read it slowly to avoid errors. (Participant 3) |
|  |  | Overall impression,  Understand the meaning,  Quick, ignore | 4 | I'll have an overall impression after reading it. (Participant 5) |
|  |  | Normally, depend on, rely on | 3 | It depends on how carefully you read it. (Participant 12) |
| Appropriate Length | Long translation of mental health knowledge makes the participants less patient. | Patient, patience, concentrate | 8 | Yes, if the content is too long, I will lose patience and stop reading. (Participant 15) |
|  |  | Useful, necessary, beneficial | 3 | I'm not willing to spend too much time on information that isn't interesting or beneficial to me. (Participant 2) |
|  | The participants would not like to read a long translation they are not interested in. | Interest, interested, interesting | 11 | Unless I am very or highly interested in it. I will find it boring to read and will stop. (Participant 14) |
|  |  | Attract, attractive, eye-catching | 6 | It is indeed not attractive enough to me. It is not attractive enough. I think two pages may be the upper limit for content that I am not very interested in. (Participant 9) |
|  | Concise translation is preferred for digital reading. | phone | 2 | Ah, if you read it on your phone, I feel that about 4 pages on your phone is suitable, but the content should be more concise. (Participant 5) |
|  |  | Wechat, public account tweet, social media | 3 | About three pages, or WeChat can load all at once. (Participant 13) |
|  |  | Short video | 3 | In this era of short videos, everyone wants to watch something quickly. (Participant 11) |
|  |  | Fragmented reading | 2 | Although there's some information on one or two pages, it's still fragmented reading, so I'll probably just glance at it, maybe just look at the subheadings or something, and only read the parts that interest me. (Participant 16) |
|  | The length of translation influences the reading strategy and information processing. | Glance, general understanding, neglect | 4 | When reading this four-page essay, the reader may prioritize the subject that interests him. For example, if I am aware that I have a problem with resilience, when I read this essay, I might neglect the introduction on resilience. (Participant 9) |
|  |  | section | 7 | The key is to control the reading time of each section. (Participant 7) |
| Combination of Pictures and Text | The combination of text and picture makes participants’ reading fast and clear. | Clear, concise, easier | 6 | If you want to be concise and clear, I believe you should merge images with words. (Participant 11) |
|  |  | Quickly, rapidly, time, faster | 6 | If it includes pictures and text, I can quickly grasp the main points. (Participant 3) |
|  | Pictures fit the participants’ fragmented reading habit. | Fragmented time, all the time, at any time | 3 | Yes, I think text plus pictures is more suitable for reading in fragmented time. (Participant 13) |
|  |  | Convenient, limited space | 5 | Maybe pictures are better on mobile phones because there is limited space. (Participant 5) |
| Video | The participants felt that videos are more convenient and easier to understand. | Easier, better, firmly, convenient | 6 | This type of procedure must be seen so that I can remember it more firmly and comprehend it better. (Participant 18) |
|  | Videos Create Stimulus and Passive Reception is Preferred by CISs. | capture, appealing, stimulus | 3 | Your visual response is a prevalent stimulus. (Participant 9) |
|  |  | Attention, impression, focus | 3 | You also get a visual impression from text. However, you are asked to see it, and you do not read it aloud. You can't hear it with your ears; instead, you just think about it. (Participant 9) |
|  |  | Active, actively, semi-active | 3 | I prefer videos because text is an active reading process, and unless I really want to read, I won't. Pictures are semi-active because I can look at some graphics and quickly understand their meaning. (Participant 19) |
|  |  | Passive, passively | 2 | I can absorb them passively. (Participant 15) |
| The Former Part | The participants would like to follow the instructions if the former part is interesting. | Interest, interesting, interested | 7 | Yes. This is more of a matter of interest. (Participant 7) |
|  |  | Curious, curiosity | 2 | I am curious about it. (Participant 13) |
|  | The participants didn’t realize they need the knowledge in the translation. | Need, not necessary | 10 | Because I don’t think it is necessary. (Participant 15) |
|  |  | Problem, difficulty, difficult | 3 | To put it another way, if I were in an incredibly difficult circumstance right now and saw someone's advice below, I might read it. (Participant 16) |
|  |  | Helpful, necessary, relevant | 4 | To be honest, I don't have any mental problems in this area now, so I will not read them. If I have, it should be very helpful. (Participant 19) |
| The Latter Part | The participants prefer direct, clear, and new instructions. | Too much information | 1 | And there's simply too much information. I don't want to read it again after finishing it. I don’t want to read it anymore. (Participant 15) |
|  |  | Latest, timely | 2 | So, maybe I will learn more about some of the latest topics. (Participant 9) |
|  |  | Convenient, directly | 2 | Then it's too hard because it doesn't provide a direct help method, and I have to search the website again. (Participant 10) |

Subtheme: organizational statement;

Code: Code extracted from semi-structured interview;

N: Number of instances
